# Re: Stroke Action Plan for Europe 2018–2030 (SAP-E): mid-term review and update

**DOI:** 10.1093/esj/aakag056

**Published:** 2026-06-08

**Authors:** Sandy Middleton, Christopher Levi, N Wah Cheung, Simeon Dale

**Affiliations:** Nursing Research Institute, St Vincent’s Health Network Sydney, St Vincent’s Hospital Melbourne and Australian Catholic University, Sydney, NSW, Australia; John Hunter Health and Innovation Precinct, Newcastle, NSW, Australia; Department of Medicine, University of Newcastle, Newcastle, NSW, Australia; University of Sydney, Sydney, NSW, Australia; Department of Diabetes & Endocrinology, Westmead Hospital, Sydney, NSW, Australia; Nursing Research Institute, St Vincent’s Health Network Sydney, St Vincent’s Hospital Melbourne and Australian Catholic University, Sydney, NSW, Australia

**Keywords:** Temperature, Hyperglycaemia, Guidelines

Dear Professor Lees,

We wish to respond to the Stroke Action Plan for Europe 2018–2030 (SAP-E) mid-term review published on 19 January 2026 in the *European Stroke Journal*. The recommendation that: “Glycaemic control in non-diabetic patients and temperature control should not be delivered to improve outcomes after stroke” (p. 11)^1^ is not supported by contemporary evidence, is based on outdated sources, and risks undermining high-value, low-cost stroke-unit care.

The new SAP-E statement misinterprets evidence and oversimplifies nuanced European Stroke Organisation (ESO) guideline recommendations for glycaemia^2^ and temperature management.^3^ The ESO guidelines do not recommend withholding glycaemic or temperature management; rather, they align with current international guidelines (Australian, American and Canadian) that advise against routine induction of hypothermia, tight glycaemic control (glucose 80–130 mg/dL [4.4–7.2 mmol/L]),^2^ and prophylactic antipyretic therapy in normothermia.^3^

Evidence supports protocolised fever and glycaemic management. The Quality in Acute Stroke Care cluster randomised controlled trial demonstrated that protocols to manage Fever, hyperglycaemia (Sugar) and Swallowing dysfunction (FeSS) in the first 72 h, reduced death and dependency at 90 days by 15.7% (NNT = 6.4).^4^ This improvement was accompanied by lower mean temperatures, lower mean glucose and improved swallow screening.^4^ Importantly, the FeSS Protocols do not advocate tight glycaemic control but promote treatment of major episodes of hyperglycaemia (glucose > 180 mmol/L [10 mmol/L]); similarly, the fever component does not recommend prophylactic antipyretic use but treatment of episodes of fever (*>*37.5 °C).

Modern trials refine, rather than invalidate glycaemic management supporting a moderate, safe glucose target, consistent with current ESO^2^^,^^3^ and international guideline recommendations. Importantly, many “non-diabetic” stroke patients may, in fact, have undiagnosed diabetes. A 2025 meta-analysis showed that among patients hospitalised with stroke, 15.3% had a new diagnosis of diabetes.^5^ Routine glucose monitoring is required to identify these patients.

Contemporary guidance is clear: there should be no hesitation in treating a severely hyperglycaemic patient simply because diabetes has not yet been diagnosed.^2^ Post-stroke hyperglycaemia, regardless of the presence of known diabetes, is strongly associated with poorer functional outcomes and increased mortality,^5^ further strengthening the rationale for monitoring and treatment. These findings support continued monitoring and treatment of hyperglycaemia within safe glucose targets in all stroke patients.

In relation to fever management following stroke, while routine fever prophylaxis is not recommended, active fever detection, investigation and treatment remain core principles of supportive stroke care.^3^

The current SAP-E recommendation regarding glucose and temperature management risks being interpreted as an instruction to withhold treatments that are essential components of high-quality stroke-unit care. This conflicts with strong real-world evidence and contemporary guideline positions.^2^^,^^3^ We respectfully ask the authors to consider revising their statement to be more consistent with European and international guidelines:

Routine prophylactic antipyretic therapy, induced hypothermia, and intensive glycaemic control are not recommended to improve outcomes after stroke. However, stroke units should continue protocol-based detection and treatment of fever and hyperglycaemia^1^ (while avoiding hypoglycaemia) as part of standard stroke care.

We thank the SAP-E authors for their substantial work and welcome continued collaboration to ensure European stroke-unit practice remains aligned with high-quality evidence.
